# Immune checkpoint inhibitors in infectious diseases: therapeutic reinvigoration, immunopathology, and precision targeting

**DOI:** 10.3389/fimmu.2026.1894437

**Published:** 2026-08-03

**Authors:** Yue Cao, Chenxi Zhao, Yanying Liang, Xvrong Li, Zenghui Zhang, Qingluan Yang, Lingyun Shao

**Affiliations:** 1 Department of Infectious Diseases, Shanghai Key Laboratory of Infectious Diseases and Biosafety Emergency Response, National Medical Center for Infectious Diseases, Huashan Hospital, Fudan University, Shanghai, China; 2 Shanghai Sci-Tech Inno Center for Infection & Immunity, Shanghai, China; 3 Key Laboratory of Medical Molecular Virology (MOE/MOH), Shanghai Medical College, Fudan University, Shanghai, China

**Keywords:** chronic viral infection, host-directed therapy, immune checkpoint inhibitor, immunopathology, infectious diseases, sepsis, T-cell exhaustion, tuberculosis

## Abstract

Immune checkpoint inhibitors (ICIs) have transformed cancer therapy by releasing inhibitory pathways that restrain antitumor T-cell responses. Their success has renewed interest in checkpoint blockade for infectious diseases, particularly chronic viral infections, sepsis-associated immunosuppression, and refractory opportunistic infections. In these settings, immune checkpoints may contribute to pathogen persistence by limiting effector function, but they also restrain tissue injury, cytokine excess, and immune-mediated organ damage. This dual role makes translation fundamentally different from oncology: the goal is not maximal immune activation, but controlled immune recalibration. Evidence is strongest for chronic viral infections and selected oncology-adjacent cohorts, whereas infection-directed trials remain early and heterogeneous. A precision framework based on pathogen burden, immune phenotype, tissue vulnerability, biomarker feasibility, safety monitoring, and implementation context is therefore essential before ICIs can be responsibly developed as host-directed therapies for infectious diseases. This review synthesizes the rationale, evidence, and safety considerations for ICIs in infectious diseases. We discuss chronic viral infections, including human immunodeficiency virus (HIV), hepatitis B virus (HBV), hepatitis C virus (HCV), and Epstein-Barr virus (EBV), and highlight EBV as a bridge between persistent viral infection, immune surveillance, lymphoproliferation, and cancer immunotherapy. We also examine tuberculosis, sepsis, fungal infections, and parasitic diseases, where checkpoint pathways may restore host defense or amplify immunopathology. Finally, we propose a precision-immunotherapy framework integrating pathogen biology, immune phenotype, tissue risk, biomarker feasibility, access, and trial design.

## 1 Introduction

The clinical success of immune checkpoint inhibitors (ICIs) in oncology has reshaped modern immunology. By targeting inhibitory pathways such as PD-1/PD-L1 and CTLA-4, ICIs can restore effector T-cell activity and generate durable tumor control in malignancies that were previously difficult to treat (1, 2). This therapeutic paradigm has encouraged investigators to ask whether similar strategies might be used to treat infectious diseases, especially chronic infections characterized by persistent antigenic stimulation and impaired T-cell function (3, 4).

Chronic viral infections such as human immunodeficiency virus (HIV) and hepatitis B virus (HBV) are associated with sustained expression of inhibitory receptors on pathogen-specific T cells, reduced proliferative capacity, impaired cytokine production, and failure to eradicate infected cells. Similar pathways may operate in tuberculosis, sepsis-associated immunosuppression, fungal infections, and parasitic diseases. These observations have led to the hypothesis that ICIs may restore antimicrobial immunity in selected patients.

In cancer, the therapeutic goal is usually to overcome immune tolerance to abnormal or transformed cells. In infectious diseases, however, the host must eliminate or contain replicating pathogens while preserving tissue integrity. Inhibitory checkpoint pathways may prevent organ damage during acute infection, maintain granuloma structure in TB, limit hepatic injury in HBV, and temper cytokine excess in sepsis. The same pathways may also contribute to viral persistence, immune paralysis, or failure to clear infected cells. Thus, checkpoint blockade in infection cannot be judged only by whether it increases immune activation; it must be evaluated by its net effect on pathogen control, tissue injury, and clinical outcomes.

We develop a disease- and mechanism-oriented framework for ICIs in infectious diseases. We argue that their future lies in precision immune modulation rather than broad antimicrobial stimulation. Responsible development will require identifying reversible immune dysfunction, defining when checkpoint pathways are protective, and determining how blockade should be combined with antimicrobial therapy, antiviral therapy, vaccination, cellular therapy, or anti-inflammatory strategies.

**Figure 1:**
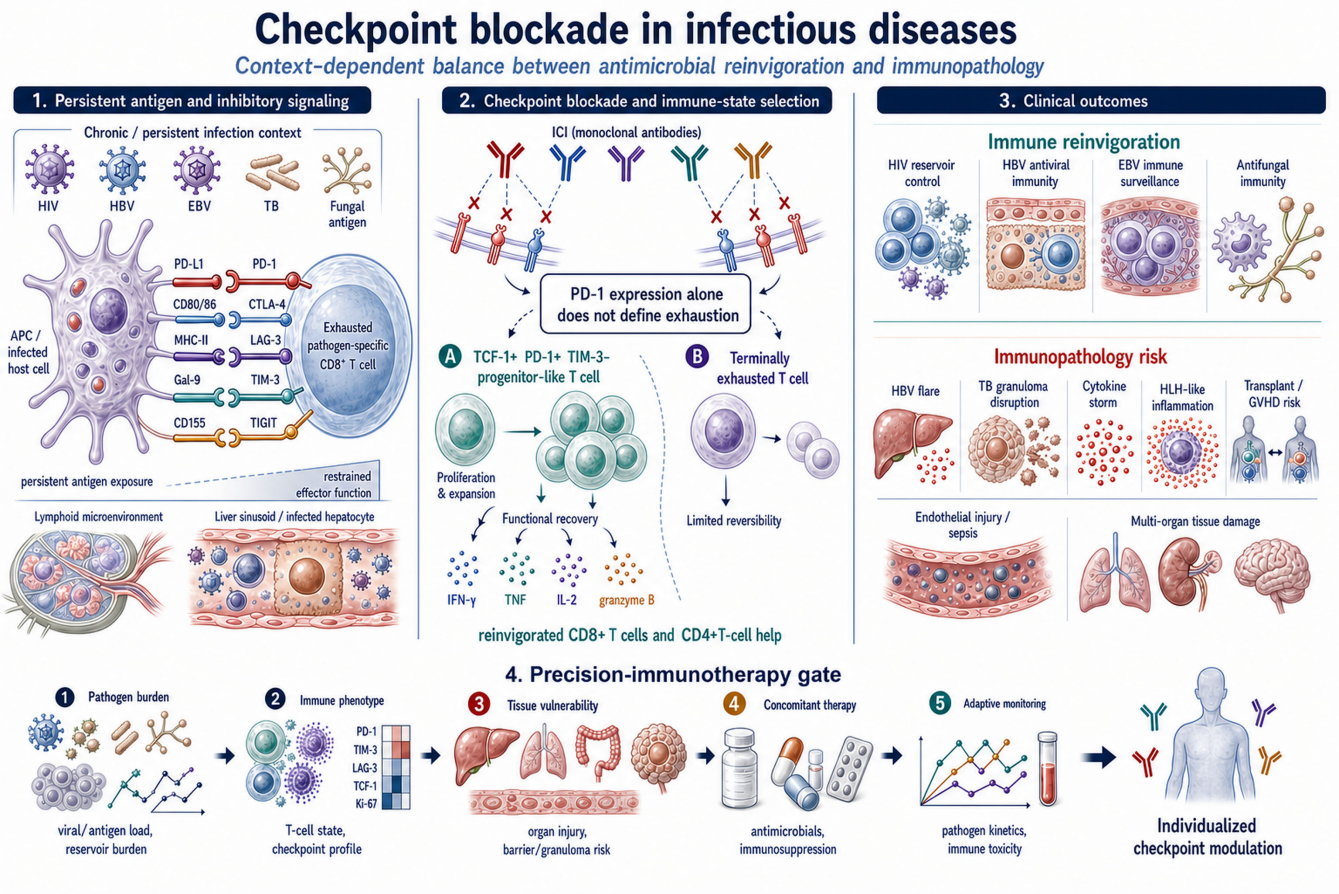
Context-dependent immune consequences of checkpoint blockade in infectious diseases. Schematic model showing how checkpoint blockade may shift infectious-disease immunity toward pathogen control or inflammatory injury according to pathogen burden, T-cell differentiation state, tissue vulnerability, and treatment context. Persistent or recurrent antigen exposure establishes inhibitory immune synapses between antigen-presenting or infected host cells and pathogen-specific T cells through PD-1/PD-L1, CTLA-4/CD80/86, LAG-3/MHC-II, TIM-3/Gal-9, TIGIT/CD155, and related pathways. Immune checkpoint blockade can restore effector programs in selected T-cell states, including expansion of TCF-1^+^ PD-1^+^ TIM-3^-^ progenitor-like T cells, reinvigoration of CD8^+^ T cells, and support of CD4^+^ T-cell help; however, PD-1 expression alone should not be interpreted as synonymous with irreversible exhaustion. Potential benefits include improved control of HIV reservoirs, HBV-infected hepatocytes, EBV-infected lymphocytes, and refractory fungal infection. In high-risk contexts, the same intervention may amplify HBV flare, TB granuloma disruption, cytokine storm, HLH-like inflammation, endothelial injury during sepsis, or transplant/GVHD-related complications. The precision-immunotherapy gate summarizes the variables that should guide individualized checkpoint modulation: pathogen burden, immune phenotype, tissue vulnerability, concomitant therapy, and adaptive monitoring.

**Figure 2:**
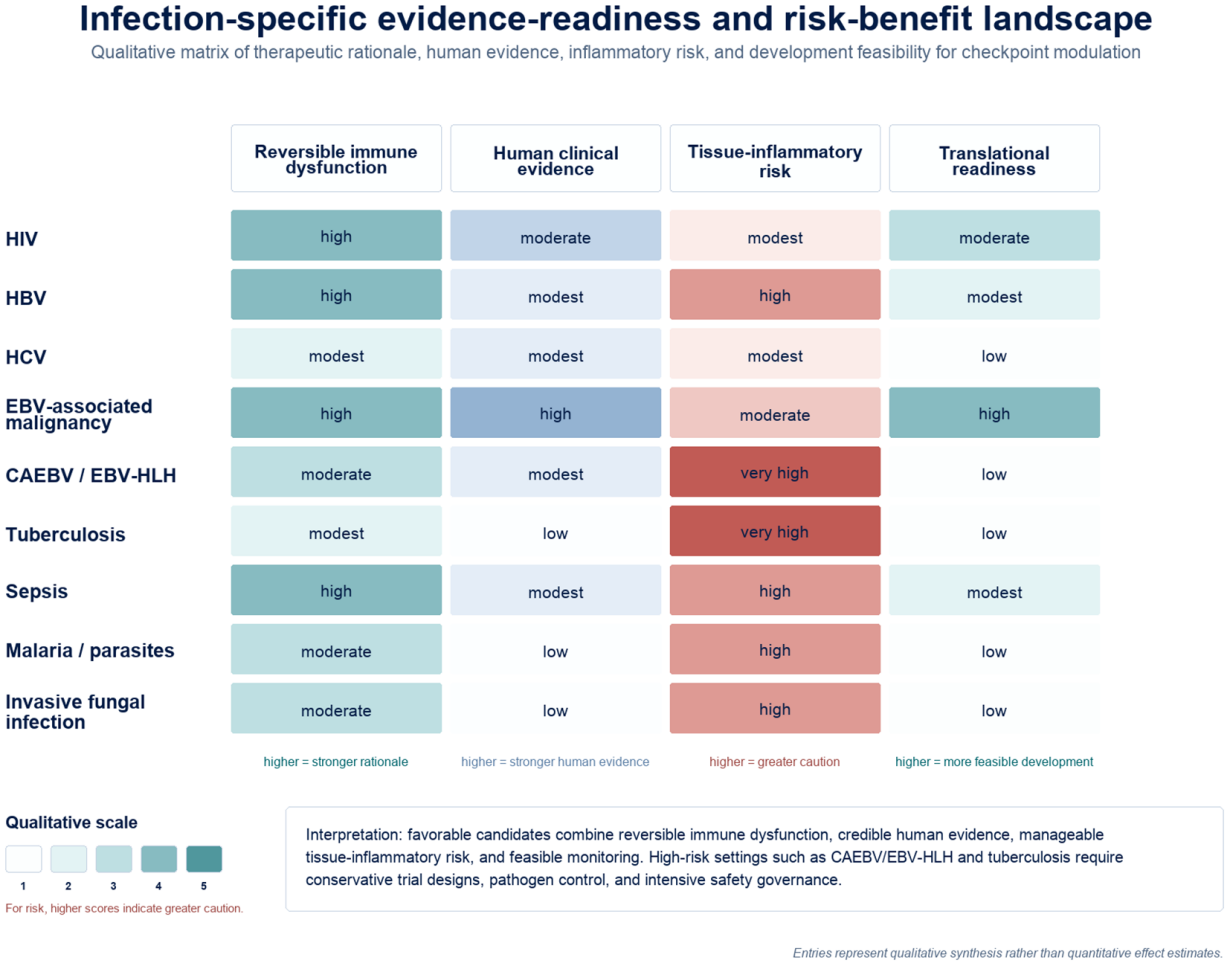
Infection-specific evidence-readiness and risk-benefit landscape. A qualitative heatmap matrix comparing major infectious-disease settings across therapeutic rationale, human clinical evidence, tissue-inflammatory risk, and translational readiness. Legend: The matrix summarizes infection-specific considerations for checkpoint modulation using four qualitative dimensions: reversible immune dysfunction, human clinical evidence, tissue-inflammatory risk, and translational readiness. Higher scores for reversible immune dysfunction, human evidence, and translational readiness indicate stronger therapeutic rationale, greater supporting evidence, and more feasible development, respectively; higher tissue-inflammatory risk indicates greater need for caution. HIV and EBV-associated malignancy show comparatively favorable translational profiles, whereas CAEBV/EBV-HLH, tuberculosis, sepsis, malaria or parasitic infection, and invasive fungal infection require more conservative trial designs because inflammatory or tissue-related hazards may outweigh immune-reinvigoration potential. Entries represent qualitative synthesis rather than quantitative effect estimates.

**Figure 3:**
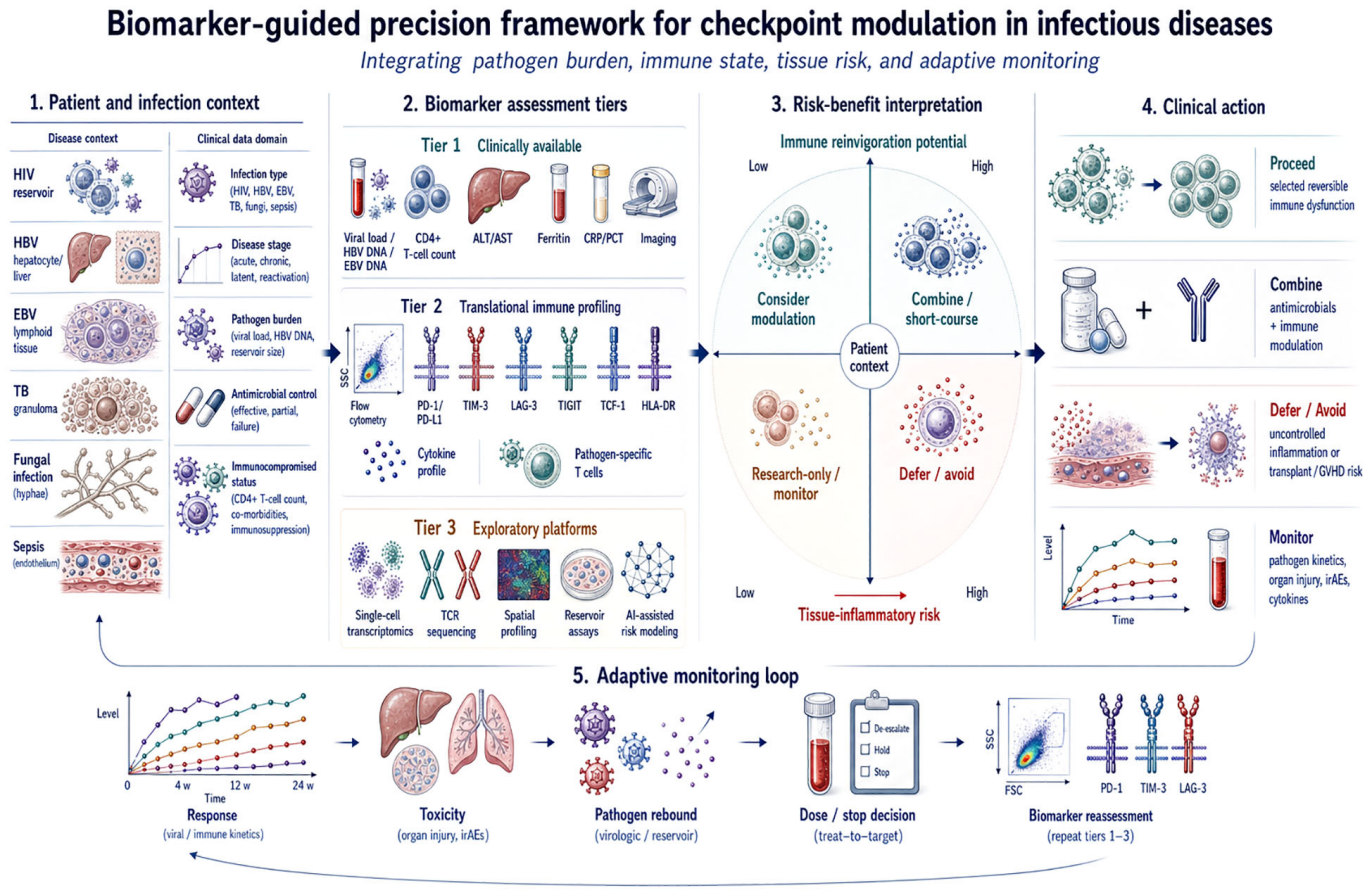
Biomarker-guided precision framework for checkpoint modulation in infectious diseases. A clinical-translational framework integrating patient and infection context, biomarker assessment tiers, risk-benefit interpretation, clinical action, and adaptive monitoring. The candidate use of checkpoint modulation in infectious diseases should be guided by pathogen and host context rather than pathogen diagnosis alone. Clinically available markers, including viral load, HBV DNA, EBV DNA, CD4^+^ T-cell count, liver enzymes, ferritin, CRP/PCT, and imaging, support initial assessment. Translational immune profiling can refine interpretation through checkpoint-marker panels, cytokine profiling, flow cytometry, and pathogen-specific T-cell assays, whereas exploratory platforms such as single-cell transcriptomics, TCR sequencing, spatial profiling, reservoir assays, and AI-assisted risk modeling may inform trial design. Risk-benefit interpretation should weigh immune reinvigoration potential against tissue-inflammatory risk to determine whether checkpoint modulation should be considered, combined with antimicrobial therapy, restricted to research-only monitoring, or deferred/avoided. The adaptive monitoring loop follows response, toxicity, pathogen rebound, dose/stop decisions, and biomarker reassessment, allowing iterative adjustment of individualized checkpoint modulation.

**Figure 4:**
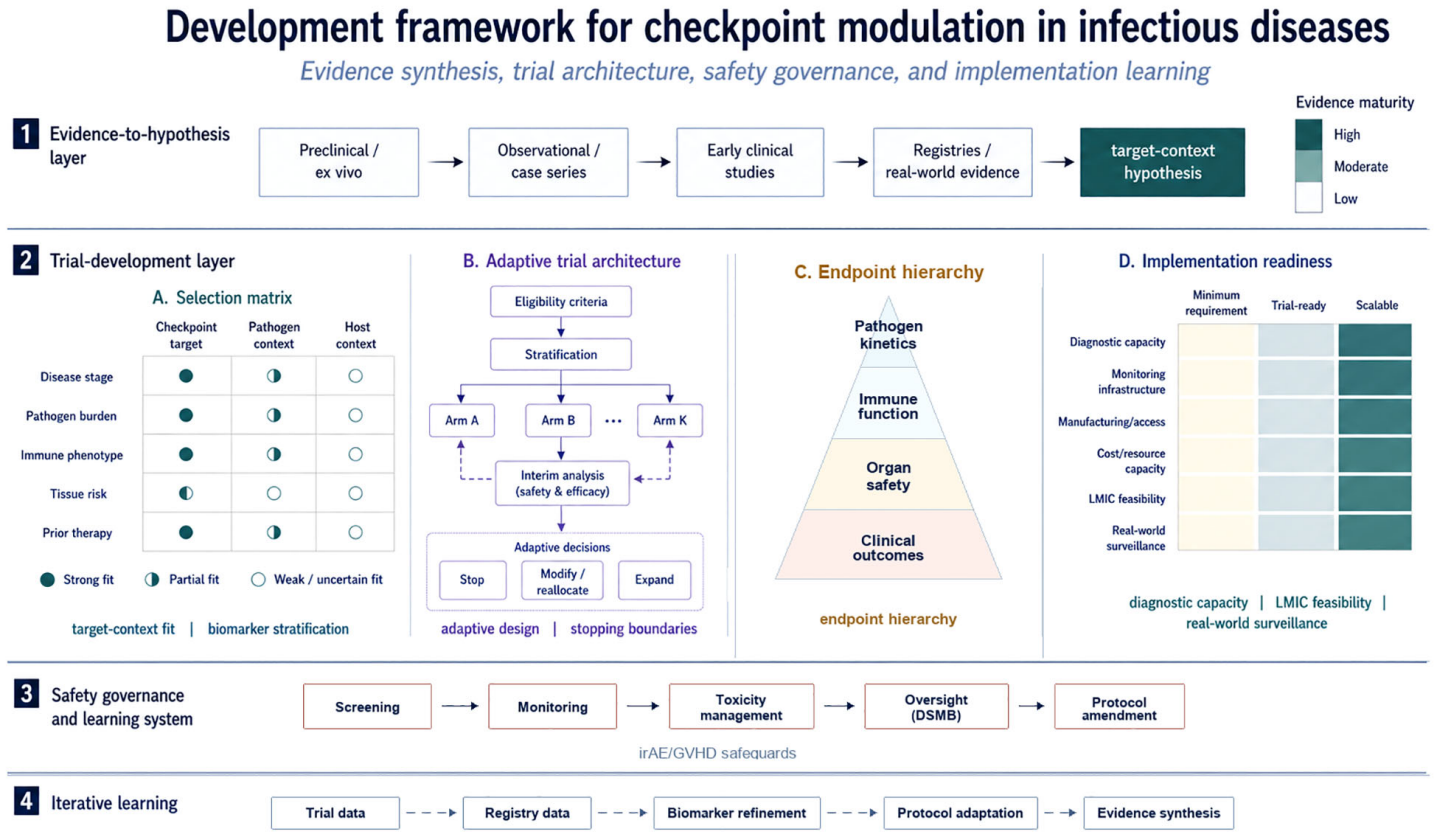
Development framework for checkpoint modulation in infectious diseases. A conceptual framework for translating checkpoint modulation in infectious diseases from evidence synthesis to biomarker-informed trial design, safety governance, implementation readiness, and iterative learning. Legend: This framework separates translational development from the mechanistic and infection-specific risk-benefit considerations shown in earlier figures. Evidence synthesis should define the maturity of preclinical, ex vivo, observational, early clinical, registry, and real-world data before a target-context hypothesis is advanced. Trial development should then integrate target-context fit, biomarker stratification, adaptive design, stopping boundaries, and a hierarchical endpoint structure that includes pathogen kinetics, immune function, organ safety, and clinical outcomes. Implementation readiness should be assessed through diagnostic capacity, monitoring infrastructure, manufacturing and access, cost and resource capacity, LMIC feasibility, and real-world surveillance. Safety governance and iterative learning link screening, monitoring, toxicity management, oversight, protocol amendment, registry data, biomarker refinement, and subsequent evidence synthesis.

The conceptual figures in this manuscript summarize the context-dependent biology of checkpoint blockade, infection-specific translational readiness, biomarker-guided patient selection, and the development pathway for clinical translation (**Figures 1**–**4**).

Given the heterogeneity and early-stage nature of this field, the literature was synthesized narratively rather than through meta-analysis or formal evidence grading. PubMed/MEDLINE, Web of Science, Google Scholar, ClinicalTrials.gov, and reference lists of relevant articles were searched for English-language mechanistic, translational, and clinical studies published up to July 2026. The review focused on literature addressing immune checkpoint biology, T-cell exhaustion, immunopathology, host-directed therapy, safety monitoring, and biomarker development in infectious diseases.

## 2 Immune checkpoints in infection: from physiological restraint to pathological exhaustion

Immune checkpoints are inhibitory pathways that calibrate immune activation. They prevent excessive responses to self-antigens, limit inflammation after antigen clearance, maintain tolerance at barrier surfaces, and restrain immune-mediated tissue injury. During infection, these pathways are induced by antigen receptor stimulation, inflammatory cytokines, tissue-derived signals, and regulatory cell networks. Their biological meaning depends on timing, tissue location, pathogen burden, and the functional state of responding immune cells.

PD-1 is among the best-characterized inhibitory receptors in chronic infection. It is expressed by activated CD4^+^ and CD8^+^ T cells, B cells, natural killer (NK) cells, and subsets of myeloid cells (5, 6). Engagement by PD-L1 or PD-L2 reduces T-cell receptor signaling, cytokine production, proliferation, and cytotoxicity (7, 8). The functional role of PD-1 in T-cell exhaustion was first established in the chronic lymphocytic choriomeningitis virus model, where PD-1 blockade restored exhausted virus-specific CD8^+^ T-cell function (7). This concept was soon extended to HIV: early studies by Trautmann, Petrovas, Freeman, and colleagues showed that PD-1 is upregulated on HIV-specific CD8^+^ T cells, correlates with impaired function and disease progression, and can mark reversible dysfunction ex vivo (9–11). Related work showed that CTLA-4 expression on HIV-specific CD4^+^ T cells defines another checkpoint-associated dysfunctional state (12). Yet PD-1 can also protect infected tissues from severe inflammation (13), so high PD-1 expression should not automatically be interpreted as an indication for blockade.

CTLA-4 primarily regulates early T-cell activation by competing with CD28 for CD80/CD86 binding and by supporting regulatory T-cell (Treg) suppressive function (14, 15). In infection, CTLA-4 can limit pathogen-specific priming, but it also prevents uncontrolled activation and autoimmunity. Because CTLA-4 blockade has broad immune-activating effects, its use in infection may carry greater inflammatory risk than more targeted approaches (16).

LAG-3, TIM-3, and TIGIT frequently co-express with PD-1 on dysfunctional T cells during chronic infection. They may identify deeper exhaustion states and may also regulate NK cells, Tregs, dendritic cells, and tissue-resident lymphocytes. Their co-expression supports combined checkpoint modulation in principle, but dual or multi-checkpoint blockade may amplify toxicity (17, 18). In infectious diseases, where cytokine excess and tissue damage are central concerns, combination strategies require a narrower therapeutic window than in oncology.

A key distinction should be made between checkpoint expression, T-cell exhaustion, and therapeutic reversibility. Checkpoint expression may reflect recent activation, chronic antigen exposure, tissue adaptation, progenitor-like exhausted differentiation, or terminal dysfunction. PD-1 positivity alone is therefore insufficient to define exhaustion. In chronic viral infection, TCF-1^+^ PD-1^+^ TIM-3^-^ memory-like or progenitor exhausted CD8^+^ T cells can retain proliferative potential and generate responses after PD-1 blockade, whereas terminally exhausted populations show more limited reversibility (19, 20). This distinction is especially relevant in infectious diseases, where benefit depends on whether reversible pathogen-specific T cells remain, whether antigen load can be reduced, and whether tissue inflammation can be controlled (21–24).

### 2.1 Checkpoint biology in infection

For infectious diseases, checkpoint pathways can be understood through four overlapping functions. They act as activation brakes during acute immune responses, contribute to adaptive tolerance during persistent antigen exposure, participate in pathological exhaustion when stimulation remains high, and shape immune microenvironments by regulating antigen-presenting cells, Tregs, NK cells, B-cell help, and cytokine circuits.

This framework helps explain why the same checkpoint molecule may have opposite therapeutic implications across infections. In HIV, PD-1 expression on HIV-specific T cells and reservoir-enriched CD4^+^ T-cell subsets supports a rationale for carefully controlled PD-1 blockade. In HBV, similar exhaustion biology occurs within a liver environment where immune restoration can damage hepatocytes. In tuberculosis (TB), PD-1 signaling may restrain destructive lung inflammation. In sepsis, PD-1/PD-L1 expression may identify patients with immunoparalysis, but only after source control and antimicrobial therapy have addressed the primary infectious insult.

The translational task is not to label checkpoints as harmful or beneficial. Rather, it is to determine whether a given patient has a reversible immune defect that limits pathogen control, and whether blocking that pathway can improve host defense without disrupting tissue-protective regulation.

### 2.2 Cellular and tissue compartments of checkpoint regulation

Checkpoint biology in infectious diseases is not confined to circulating CD8^+^ T cells. Tissue-resident memory T cells, exhausted progenitor-like T cells, terminally exhausted effector cells, T follicular helper cells, Tregs, NK cells, B cells, monocytes, macrophages, dendritic cells, and stromal cells may all contribute to the net effect of checkpoint blockade (25–33). This matters because pathogen control is often anatomically compartmentalized. HIV reservoirs are enriched in lymphoid tissues and long-lived memory CD4^+^ T cells. HBV immune responses occur within a tolerogenic hepatic microenvironment (34). TB bacilli persist in granulomas where macrophage activation, necrosis, hypoxia, and tissue architecture determine outcome (35). EBV latency is shaped by B-cell biology, T/NK-cell infection in chronic active EBV disease (CAEBV), lymphoid tissue organization, and tumor microenvironmental escape (36).

Several mechanistic dimensions should therefore be considered before interpreting checkpoint expression. The first is cell lineage: PD-1 on virus-specific CD8^+^ T cells has different implications from PD-1 on T follicular helper cells, Tregs, or reservoir-enriched CD4^+^ T cells. The second is differentiation state: progenitor-like exhausted T cells may expand after PD-1 blockade, whereas terminally exhausted cells may remain poorly reversible (8, 37). The third is ligand distribution: PD-L1 expression on hepatocytes, monocytes, macrophages, endothelial cells, tumor cells, or infected lymphocytes may reflect different inflammatory cues and may produce different tissue outcomes (5). The fourth is metabolic and cytokine context: hypoxia, type I interferons, IL-10, TGF-β, adenosine, indoleamine 2,3-dioxygenase, and chronic antigen load can reinforce checkpoint-driven dysfunction (38, 39).

This broader compartmental view is especially important for non-PD-1 pathways. LAG-3 may cooperate with PD-1 to restrain exhausted T cells and may also regulate antigen-presenting cell interactions (40). TIM-3 can mark dysfunctional T cells, innate immune cells, or terminally differentiated states depending on context (41). TIGIT suppresses T-cell and NK-cell responses through the CD155/CD112 axis and may be relevant in chronic viral infection, sepsis, and virus-associated tumors; in HIV and SIV infection, TIGIT marks exhausted T cells, correlates with disease progression, and has been proposed as a target for immune restoration (42, 43). VISTA is a myeloid-enriched regulator of inflammatory tone, making it relevant to acute infection and sepsis, where monocytes, macrophages, tissue-resident myeloid cells, and gamma-delta T-cell responses shape both immune paralysis and inflammatory injury (44). CD47/SIRPα signaling provides a phagocytic checkpoint that may influence clearance of infected, stressed, or pathogen-containing cells, although broad blockade could cause hematologic toxicity and excessive innate activation. Together with BTLA, CD39/CD73-mediated adenosine signaling, and other metabolic or stromal checkpoints, these pathways argue for pathway-specific, time-limited combinations guided by pathogen burden, immune phenotype, and tissue vulnerability (45–48).

## 3 Chronic viral infections

Chronic viral infections have provided the strongest rationale for applying checkpoint blockade outside oncology. Persistent viral antigen can drive sustained inhibitory receptor expression and functional impairment of virus-specific T cells. Nevertheless, chronic viral infections differ greatly in reservoir biology, tissue tropism, immune pathology, and available antiviral therapy. These differences shape both the therapeutic promise and the risk of ICI-based strategies.

### 3.1 HIV infection

HIV infection remains a major focus of checkpoint-blockade research because antiretroviral therapy (ART) suppresses plasma viremia but does not eradicate latent reservoirs. HIV persists in long-lived CD4^+^ T cells, including immune-checkpoint-enriched subsets (49). Following the LCMV work, studies by Trautmann, Petrovas, Freeman and colleagues established PD-1/PD-L1 as a central axis of HIV-specific T-cell dysfunction and showed that blockade could enhance HIV-specific T-cell function ex vivo (9–11). CTLA-4 upregulation on HIV-specific CD4^+^ T cells was also linked to disease progression and reversible dysfunction (12). More recent work indicates that PD-1, CTLA-4, TIGIT, and LAG-3 mark CD4^+^ T-cell subsets enriched for intact proviruses, connecting checkpoint biology to both effector dysfunction and reservoir persistence (49, 50).

Checkpoint blockade may affect HIV persistence through two main mechanisms. It may restore HIV-specific CD8^+^ T-cell function, improving recognition and elimination of infected cells (51). It may also perturb latency by activating infected CD4^+^ T cells or modifying dendritic-cell and tissue microenvironments that maintain reservoirs (52–54). These mechanisms support cure-oriented strategies, but they also raise safety concerns, including generalized immune activation, viral rebound, and inflammation in tissue reservoirs.

Clinical evidence remains limited. Most human experience derives from people living with HIV who received ICIs for cancer. These studies and case series suggest that PD-1 or CTLA-4 blockade can be administered to selected patients on suppressive ART without consistently compromising virological control, and they provide important safety and immunological data (55, 56). However, oncology cohorts cannot establish HIV cure efficacy because tumor type, chemotherapy, corticosteroid exposure, ART history, reservoir size, and immune status confound interpretation. Cure-directed trials will need to test whether checkpoint modulation can reduce intact reservoir size, delay rebound during analytical treatment interruption, or improve immune control after latency reversal.

The central challenge in HIV is that immune activation is not inherently beneficial. Excessive activation may increase target-cell availability, destabilize reservoirs without clearance, or worsen inflammatory comorbidities. Future studies should therefore prioritize selected populations, short-course dosing, intensive virological monitoring, and combination strategies that pair immune reinvigoration with effector mechanisms capable of eliminating infected cells.

Another unresolved issue is anatomical access. Most clinical reservoir measurements rely on blood, but lymph nodes, gut-associated lymphoid tissue, genital tissues, and the central nervous system may respond differently to checkpoint modulation. An ICI-induced increase in HIV transcription in blood may not correspond to efficient clearance in tissues. Future studies should integrate tissue sampling when feasible, or use validated surrogate measures that better reflect anatomically distributed reservoirs. In addition, the timing of checkpoint blockade relative to therapeutic vaccination or broadly neutralizing antibody administration may determine whether latency reversal is followed by immune clearance or simply transient viral transcription.

The recognition of stem-like or progenitor exhausted T cells further refines this HIV framework. A recent study reported that depletion of plasmacytoid dendritic cells could rescue HIV-reactive stem-like CD8^+^ T cells during chronic HIV-1 infection, supporting the idea that HIV-specific immunity may include PD-1^+^ populations with retained proliferative potential rather than uniformly terminally exhausted cells (20). For HIV cure strategies, PD-1 expression should therefore be interpreted alongside TCF-1, TIM-3, antigen specificity, reservoir localization, and functional assays.

### 3.2 HBV infection

Chronic HBV infection is characterized by incomplete immune control of infected hepatocytes, persistence of covalently closed circular DNA, and variable liver inflammation and fibrosis (57). HBV-specific CD8^+^ T cells often show impaired effector function and increased inhibitory receptor expression, particularly with high antigen burden (58). These observations support the rationale for restoring antiviral immunity, especially if checkpoint modulation is combined with antigen reduction or other immunomodulatory strategies (59).

Despite this rationale, HBV presents a major safety challenge. The liver is a tolerogenic organ in which immune restraint protects against severe tissue injury (60). Reinvigorating HBV-specific T cells could enhance viral control but may also trigger hepatitis flares, hepatocyte destruction, or hepatic decompensation in vulnerable patients. This risk is especially relevant in patients with advanced fibrosis, cirrhosis, or high intrahepatic antigen burden (61).

The most plausible future for ICIs in HBV is staged combination treatment rather than long-term monotherapy. Antiviral therapy or antigen-lowering agents may first reduce the inflammatory substrate. Transient checkpoint modulation could then be used to restore antiviral effector function, guided by biomarkers of T-cell reversibility and liver safety. This model resembles cancer combination immunotherapy but requires greater caution because the principal infected tissue is a vital organ with limited tolerance for immune-mediated injury.

HBV also illustrates the gap between systemic and organ-localized immune restoration (62). Peripheral HBV-specific T-cell assays may underestimate intrahepatic immunity, whereas repeated liver biopsy is rarely feasible (63). Noninvasive biomarkers combining HBsAg quantity, HBV RNA, hepatitis B core-related antigen, alanine aminotransferase kinetics, and circulating immune phenotypes will be needed to define a safe therapeutic window.

### 3.3 HCV infection

HCV historically provided important insights into T-cell exhaustion, inhibitory receptor expression, and failure of spontaneous viral clearance (64, 65). However, the therapeutic landscape has changed dramatically with direct-acting antivirals, which cure most patients with short, well-tolerated regimens. As a result, checkpoint blockade has less obvious therapeutic relevance for HCV infection itself than for HIV or HBV (66).

Current interest in HCV and ICIs is primarily translational and safety-oriented. In patients with HCV-associated hepatocellular carcinoma or prior HCV infection, ICIs may be used for cancer therapy. Available evidence suggests that many such patients can receive checkpoint blockade, but careful monitoring of hepatic inflammation and viral kinetics remains important (67). Mechanistically, HCV continues to illustrate how viral clearance, antigen decline, and T-cell exhaustion interact (68). The HCV field also cautions that reversal of exhaustion may not be necessary when highly effective pathogen-directed therapy exists.

### 3.4 EBV infection and EBV-associated diseases

Epstein-Barr virus (EBV) occupies a distinctive position in the checkpoint-blockade landscape. EBV establishes lifelong latency, primarily in B cells, and is controlled by continuous immune surveillance involving EBV-specific CD8^+^ T cells, CD4^+^ T cells, and NK cells (69, 70). It is also linked to diverse diseases, including infectious mononucleosis, chronic active EBV disease, post-transplant lymphoproliferative disease, hemophagocytic lymphohistiocytosis, Hodgkin lymphoma, nasopharyngeal carcinoma, EBV-positive gastric carcinoma, and extranodal NK/T-cell lymphoma (71).

EBV latency programs allow infected cells to persist while limiting immune visibility. In healthy hosts, robust EBV-specific T-cell immunity constrains latent and lytic infection. However, in chronic active EBV disease and EBV-associated lymphoproliferative disorders, immune surveillance is insufficient or distorted (36). Several studies have identified expression of PD-1, PD-L1, TIM-3, LAG-3, VISTA, CD47, and other inhibitory pathways in EBV-associated immune microenvironments (72–81). In EBV-positive malignancies, viral antigens, inflammatory signaling, 9p24.1 alterations, and interferon-driven programs may contribute to PD-L1 upregulation and sensitivity to PD-1/PD-L1 blockade (82, 83).

The strongest clinical support for checkpoint blockade in the EBV field comes from EBV-associated malignancies rather than uncomplicated EBV infection. PD-1 blockade has shown activity in classical Hodgkin lymphoma, nasopharyngeal carcinoma, and other EBV-associated tumors in selected settings (52, 54, 82–87). These outcomes demonstrate that EBV-associated immune escape can be therapeutically targeted (72). However, antitumor efficacy should not be directly extrapolated to treatment of EBV infection itself. In malignancy, the goal is to restore immune pressure against transformed cells within a tumor microenvironment. In non-malignant EBV infection, the target may be a dynamic reservoir of infected lymphocytes embedded in systemic immune dysregulation.

Chronic active EBV disease is especially complex. It is not prolonged viral shedding or recurrent serological reactivation, but a severe EBV-driven lymphoproliferative disease often involving infected T or NK cells, systemic inflammation, high EBV DNA levels, organ involvement, and risk of progression to lymphoma or leukemia (87–89). Immune checkpoint pathways may participate in the dysfunctional inflammatory environment of CAEBV, and PD-1 blockade has been explored in refractory EBV-associated hemophagocytic lymphohistiocytosis (HLH) or CAEBV-related settings (90). Yet the therapeutic logic differs from that in EBV-positive cancer: in CAEBV, the infected lymphocyte itself may drive inflammation and clonal expansion. Enhancing T-cell activation could improve immune control in selected cases, but it may also worsen cytokine-mediated injury, trigger HLH-like deterioration, or complicate subsequent allogeneic hematopoietic stem cell transplantation (allo-HSCT) (91). PD-1 inhibitor exposure before allo-HSCT has been associated with heightened alloreactive immune responses in other disease settings, raising concern for increased graft-versus-host disease (GVHD) risk. PD-1 blockade in CAEBV should therefore remain investigational and should be integrated with EBV DNA monitoring, inflammatory markers, infected-cell lineage assessment, transplant planning, and GVHD risk mitigation.

EBV should therefore be framed as both opportunity and caution. It supports the biological relevance of checkpoint pathways in persistent viral immune surveillance, but it also shows why infection-associated immune activation cannot be treated as uniformly beneficial. The therapeutic question is whether checkpoint blockade targets malignant immune escape, reversible antiviral dysfunction, or a self-amplifying inflammatory lymphoproliferative process.

A practical EBV framework should begin with infected-cell lineage and disease category. EBV-positive Hodgkin lymphoma or nasopharyngeal carcinoma may be approached as virus-associated cancer with checkpoint-sensitive immune escape. Post-transplant lymphoproliferative disease is shaped by iatrogenic immunosuppression and may require reduction of immunosuppression, rituximab, cellular therapy, or selected immunotherapy. CAEBV and EBV-HLH require special caution because viral control, lymphoproliferation, cytokine storm, and HSCT timing are closely intertwined. EBV DNA kinetics, infected-cell lineage, tissue involvement, inflammatory markers, and transplant eligibility should therefore guide any checkpoint-based strategy.

### 3.5 Other chronic or persistent viral infections

Other persistent viral infections, including HPV, CMV, and herpesviruses, may involve checkpoint pathways, especially in immunocompromised hosts or virus-associated cancers. HPV-associated malignancies are already part of the clinical ICI landscape, but whether checkpoint blockade alters HPV infection itself remains uncertain (92). CMV-specific T cells can show differentiated or exhausted phenotypes in transplant recipients, but broad checkpoint blockade may be hazardous in these populations (93, 94). Across persistent viral infections, the key question is whether inhibitory pathways primarily mark reversible antiviral dysfunction, compensatory tissue protection, or cancer-associated immune escape.

## 4 Tuberculosis and other intracellular infections

Tuberculosis (TB) is one of the clearest examples of checkpoint biology as a double-edged sword. Mycobacterium tuberculosis persists within granulomatous lesions that reflect a dynamic equilibrium between antimicrobial immunity and inflammatory containment. T cells, macrophages, cytokines, and tissue architecture cooperate to restrict bacterial growth, but excessive immune activation can destroy lung tissue and promote transmission (95).

PD-1 expression is observed on T cells during TB, and PD-1/PD-L1 interactions may suppress antimycobacterial effector responses in some contexts (96). This has generated interest in checkpoint pathways as host-directed therapeutic targets. However, animal studies and clinical observations caution that PD-1 may protect against TB by restraining damaging inflammation (97, 98). Cases of TB reactivation or worsening during cancer ICI therapy reinforce this concern.

These findings distinguish TB from a simple exhaustion-reversal model. The desired intervention is restoration of effective macrophage-directed immunity without damaging granuloma structure or lung tissue (99). If explored, checkpoint-based host-directed therapy for TB should be limited to highly selected settings, combined with effective antimycobacterial therapy, and monitored for bacterial burden, cavitation, and inflammatory worsening.

The granuloma is the key unit of TB immunobiology. Within granulomas, PD-1/PD-L1 interactions may influence T-cell activation, macrophage function, necrosis, bacterial replication, and tissue remodeling (100, 101). Peripheral biomarkers may therefore fail to capture the biology most relevant to disease control. Spatial immunology, tissue imaging, and careful phenotyping will be needed before checkpoint modulation can be considered rational in TB.

Other intracellular pathogens, such as non-tuberculous mycobacteria, Listeria, Salmonella, and certain fungal organisms, may similarly induce checkpoint pathways during persistent infection. However, the evidence base is smaller, and the risk-benefit balance is uncertain. In immunocompromised hosts, checkpoint blockade may theoretically augment cellular immunity, but it may also provoke inflammatory syndromes or unmask subclinical infection. The field should avoid assuming that all intracellular infections will benefit from PD-1 blockade.

## 5 Sepsis and acute bacterial infections

Sepsis is a life-threatening syndrome of organ dysfunction caused by a dysregulated host response to infection. It is often conceptualized as early hyperinflammation followed by immunosuppression, although in practice these states overlap (102). Many patients show simultaneous features of cytokine activation, endothelial injury, lymphocyte apoptosis, reduced antigen presentation, impaired phagocyte function, and susceptibility to secondary infections (103, 104).

The rationale for checkpoint blockade in sepsis is based on the immunosuppressive phenotype observed in a subset of patients. Increased PD-1 expression on T cells, increased PD-L1 expression on monocytes and other antigen-presenting cells, reduced monocyte HLA-DR expression, lymphopenia, and impaired cytokine production have been associated with poor outcomes (105–107). Preclinical studies suggest that PD-1/PD-L1 blockade can improve immune cell survival, restore cytokine responses, enhance bacterial clearance, and improve survival in selected models (108).

Translation in human sepsis is challenging because sepsis is not a single disease but a syndrome shaped by pathogen, infection site, host background, source control, and treatment timing. A phase 1b randomized, placebo-controlled study of the anti-PD-L1 antibody BMS-936559 and a separate phase 1b randomized study of nivolumab showed acceptable safety and pharmacodynamic activity in selected patients with severe sepsis or septic shock (108, 109). These trials support feasibility, but they were not powered to establish mortality benefit or define optimal enrichment strategies.

The most defensible strategy is biomarker-guided, short-course checkpoint modulation in patients with demonstrable immune paralysis. Potential selection markers include persistent lymphopenia, low monocyte HLA-DR, high PD-1/PD-L1 expression, impaired ex vivo cytokine responses, secondary infection, and failure to recover immune function after source control and antibiotics. Even in this population, checkpoint blockade should be viewed as adjunctive immunomodulation rather than antimicrobial therapy.

Sepsis immunology changes rapidly. A single baseline PD-L1 value may not distinguish a patient entering immune paralysis from one recovering from early inflammation (110, 111). Dynamic monitoring may be more informative than static enrollment markers. Serial monocyte HLA-DR, absolute lymphocyte count, ex vivo TNF or IFN-γ response, cytokine trajectories, and secondary infection patterns may help identify patients with persistent immunosuppression and lower inflammatory risk.

## 6 Parasitic, fungal, and neglected infections

Parasitic infections provide additional evidence that checkpoint pathways can regulate both pathogen persistence and immunopathology. Many parasitic diseases are chronic, antigenically complex, and tissue-compartmentalized. They often require coordinated CD4^+^ T-cell help, cytotoxic T-cell activity, antibody production, macrophage activation, and regulatory mechanisms that limit tissue injury (112). This makes them biologically attractive but clinically difficult settings for checkpoint modulation.

In malaria, inhibitory receptors are associated with impaired T-cell and B-cell responses, but severe disease also reflects inflammatory damage, endothelial activation, and tissue-specific pathology (113, 114). Experimental studies have shown that PD-1, PD-L1, and LAG-3 pathways can limit protective T-cell help and antibody responses during Plasmodium infection, and combined blockade of PD-L1 and LAG-3 can accelerate parasite clearance in selected mouse models (4, 115). These data are mechanistically important because they link checkpoint pathways to defective antimalarial immunity. However, they should not be extrapolated directly to clinical blockade. Malaria pathogenesis depends on parasite species, life-cycle stage, host age, prior exposure, pregnancy status, vascular inflammation, and organ involvement. In cerebral malaria or severe inflammatory disease, releasing inhibitory pathways could worsen immunopathology.

Leishmaniasis, toxoplasmosis, Chagas disease, and other protozoan infections also involve chronic antigen exposure and T-cell dysfunction (116–118). In some models, blockade of PD-1 or related pathways improves effector function, whereas in others it increases inflammatory injury or fails to overcome parasite persistence (119). The diversity of parasite biology argues for disease-specific development rather than broad extrapolation from viral exhaustion models.

Fungal infections are an emerging area of interest because many invasive mycoses occur in hosts with impaired cellular immunity. Enhancing T-cell, macrophage, or neutrophil-supporting circuits could theoretically improve control of Candida, Aspergillus, Cryptococcus, Mucorales, or Pneumocystis. However, evidence is largely limited to mechanistic studies, extrapolation from oncology, and case reports (119–121). The diversity of fungal pathogens and host immune defects makes empirical checkpoint blockade inappropriate outside exceptional circumstances.

For fungal disease, the most realistic near-term role of checkpoint blockade is not empirical treatment of mycoses but individualized adjunctive immunotherapy in refractory cases with evidence of impaired cellular immunity, effective antifungal therapy already in place, and careful monitoring for inflammatory deterioration. Translational studies should also determine whether checkpoint blockade acts directly on antifungal T cells, indirectly through myeloid function, or primarily by remodeling inflammatory networks.

Neglected infections should be included in future checkpoint research because they often involve chronic antigen exposure, tissue compartmentalization, and immune regulation. Yet access, trial infrastructure, and safety monitoring remain major barriers. A globally relevant agenda should prioritize low-risk biomarkers, affordable combination strategies, and endpoints that reflect local disease burden rather than oncology-derived response metrics alone.

## 7 Clinical evidence and translational landscape

The clinical evidence base for ICIs in infectious diseases remains uneven. The strongest human data derive indirectly from patients with chronic viral infections who receive ICIs for cancer. These data are useful for safety and mechanistic inference but do not establish efficacy against infection. Direct infection-focused trials remain small, early-phase, or disease-specific.

Disease-specific evidence, checkpoint targets, combination strategies, unresolved questions, biomarker considerations, and representative clinical studies are summarized in **Tables 1**–**6**.

**Table 1:** Evidence map for checkpoint blockade in selected infectious diseases.

Infection setting	Main checkpoint pathways	Evidence base	Potential benefit	Main risk	Key references
HIV	PD-1/PD-L1, CTLA-4, TIGIT, LAG-3	Ex vivo studies, SIV models, cancer cohorts, early-phase trials	Enhanced HIV-specific immunity, latency perturbation, possible reservoir effects	Immune activation, uncertain reservoir clearance, inflammatory toxicity	(52–56, 76, 128, 129)
HBV	PD-1/PD-L1, CTLA-4, TIM-3	Ex vivo studies, early clinical exploration, cancer cohorts	Restoration of HBV-specific T-cell function, functional cure strategies	Hepatitis flare, hepatocyte injury, liver failure in high-risk patients	(59, 77, 131, 132, 134, 163–165)
HCV	PD-1/PD-L1	Mechanistic studies, cancer safety data	Limited direct role due to effective DAAs	Hepatic inflammation, limited incremental value	(67, 134)
EBV	PD-1/PD-L1, TIM-3, LAG-3, CD47 and others	EBV-associated cancer trials, CAEBV reports, mechanistic studies	Antitumor immunity in EBV-associated malignancy, possible antiviral immune restoration	HLH-like inflammation, lymphoproliferation, tissue injury, possible GVHD risk if PD-1 blockade precedes allo-HSCT	(52, 54, 72–88, 90–92, 126, 127, 129, 130, 138, 165, 166)
TB	PD-1/PD-L1	Animal models, case reports, pharmacovigilance	Theoretical host-directed immune enhancement	Reactivation, granuloma disruption, lung immunopathology	(85, 92, 96–98, 135)
Sepsis	PD-1/PD-L1	Preclinical studies, biomarker studies, early clinical trials	Reversal of immune paralysis, improved secondary infection control	Worsened inflammation, timing errors, heterogeneous response	(75, 77, 78, 81, 105–109, 138, 139, 167)

**Table 2:** Major immune checkpoint pathways in infectious diseases.

Pathway	Main immune role	Infection relevance	Therapeutic caution	Key references
PD-1/PD-L1	Limits TCR signaling and effector function	T-cell exhaustion, sepsis immune paralysis, viral persistence	May prevent tissue damage; blockade may reactivate latent infection	(3, 4, 7, 8, 52–56, 59, 85, 96–98, 105–109, 167)
CTLA-4	Regulates priming and Treg function	T-cell activation threshold, HIV reservoir studies	Broad immune activation and higher inflammatory toxicity	(1, 2, 53, 54, 128, 129)
LAG-3	Regulates exhausted T cells and antigen-presenting interactions	Chronic viral infection, EBV-associated malignancy, malaria	Often co-expressed with PD-1; combination blockade may increase toxicity	(3, 4, 72, 73, 113, 124, 125)
TIM-3	Regulates exhausted and innate immune cells	Viral persistence, EBV, sepsis	Context-dependent; may mark terminal dysfunction	(3, 4, 72, 73, 124, 125)
TIGIT	Regulates T and NK cells	HIV, chronic viral infection, tumor-virus interface	Clinical infectious-disease role remains early	(3, 52, 76, 124–126)
VISTA	Regulates myeloid and T-cell inflammatory tone	Sepsis, chronic inflammation, tumor-virus microenvironments	Biology remains context-dependent; limited infection-directed clinical data	(72, 73, 124–126, 138)
BTLA/HVEM	Maintains lymphocyte inhibitory signaling and tolerance	Chronic viral infection, T-cell regulation	May restrain immunopathology; therapeutic role remains exploratory	(3, 124, 125, 129)
CD39/CD73-adenosine	Suppresses effector cells through extracellular adenosine	Hypoxic tissues, TB granulomas, chronic viral infection, tumors	Targeting may increase inflammation in vulnerable tissues	(38, 39, 124–126)
CD47/SIRP-alpha	Limits phagocytosis and innate immune clearance	EBV-associated malignancies, myeloid regulation, intracellular infection concepts	Hematologic toxicity and broad innate activation require caution	(72, 73, 124–126)

**Table 3:** Combination strategies for future development.

Disease	Potential combination	Rationale	Key references
HIV	ICI + ART + latency reversal + therapeutic vaccine/bNAb	Pair latency perturbation with immune-mediated clearance	(52, 55, 56, 76, 128, 129)
HBV	ICI + nucleos(t)ide analog + antigen-lowering therapy + vaccine	Reduce antigen burden before immune reinvigoration	(59, 131, 132, 134, 163–165)
EBV-associated malignancy	ICI + chemotherapy/radiotherapy + EBV-specific T cells	Enhance antitumor immunity against virus-associated targets	(52, 54, 82–87, 126)
CAEBV	Cellular therapy or HSCT-centered approaches with cautious immunomodulation	Address EBV-infected T/NK cell disease and systemic inflammation; PD-1 blockade may be considered only as investigational bridging or salvage therapy before definitive HSCT	(72–81, 86, 88, 90–92, 130, 138, 165, 166)
Sepsis	ICI + source control + antibiotics + immune phenotyping	Reverse immune paralysis in selected patients	(75, 77, 78, 105–109, 138, 139, 167)
TB	ICI only with effective antimycobacterial therapy in exceptional trial settings	Avoid granuloma destabilization and inflammatory lung injury	(85, 92, 96–98, 135)

**Table 4:** Key unresolved questions for clinical translation.

Domain	Key question	Practical implication	Key references
Disease selection	Which infections contain a reversible exhausted T-cell compartment?	Prioritize mechanistic enrichment rather than broad pathogen categories	(3, 4, 37, 124, 125)
Timing	When does checkpoint expression represent harmful exhaustion rather than protective restraint?	Match dosing window to infection stage and tissue risk	(77, 78, 81, 85, 97, 98, 113, 114)
Combination therapy	Which pathogen-directed therapies should precede or accompany checkpoint blockade?	Reduce antigen burden before immune reinvigoration when possible	(55, 56, 59, 120, 121, 128, 129, 134)
Safety	Which latent infections require screening or prophylaxis before ICI exposure?	Develop infection-specific risk algorithms	(74, 77, 79, 85, 92, 98, 131–133, 135, 149, 150, 165)
Biomarkers	Can clinically feasible assays identify immune paralysis or reversible exhaustion rapidly?	Convert single-cell and transcriptomic signatures into practical tests	(37, 76–78, 107–109, 139, 167)
Endpoints	What outcome defines success: pathogen clearance, reservoir reduction, immune restoration, or survival?	Align endpoints with disease biology and patient benefit	(55, 56, 59, 76, 109, 128, 139, 167)

**Table 5:** Disease-specific biomarker and safety monitoring framework.

Disease setting	Baseline stratification	On-treatment immune monitoring	Pathogen/tissue safety monitoring	Key references
HIV	ART status, plasma HIV RNA, CD4 count, reservoir assays when available, cancer or cure-trial context	HIV-specific T-cell function, activation markers, checkpoint co-expression, inflammatory cytokines	Plasma HIV RNA, cell-associated HIV RNA/DNA, immune activation, inflammatory comorbidities	(52–56, 76, 127–129)
HBV	HBsAg, HBeAg, HBV DNA, HBV RNA/HBcrAg if available, fibrosis stage, liver reserve	HBV-specific T-cell responses, cytokine activation, intrahepatic surrogates	ALT/AST, bilirubin, INR, albumin, HBV DNA, hepatitis flare criteria	(59, 77, 131, 132, 134, 163–165)
EBV/CAEBV	EBV DNA, infected-cell lineage, lymphoma assessment, ferritin, cytopenias, organ involvement, HSCT candidacy	EBV-specific T/NK-cell function, PD-1/PD-L1 expression, cytokine profile, inflammatory activity before HSCT	EBV DNA kinetics, HLH markers, liver function, lymphoma transformation, inflammatory deterioration, GVHD surveillance after allo-HSCT	(72–81, 86, 88, 90–92, 130, 138, 165, 166)
TB	TB exposure history, IGRA/TST, imaging, sputum microbiology, disease activity	T-cell activation, macrophage/inflammatory signatures, cytokines	Sputum culture, imaging, symptoms, paradoxical reactions, lung tissue injury	(85, 92, 96–98, 135)
Sepsis	Source control status, infection site, lymphocyte count, monocyte HLA-DR, PD-1/PD-L1, secondary infection	Serial HLA-DR, ex vivo cytokine production, lymphocyte recovery, cytokine trajectory	SOFA score, secondary infections, organ dysfunction, inflammatory worsening	(75, 77, 78, 81, 105–109, 138, 139, 167)
Fungal/parasitic infections	Host immune status, pathogen burden, tissue involvement, antifungal/antiparasitic therapy adequacy	Cellular immune recovery, T-cell/myeloid checkpoint phenotype, inflammatory markers	Microbiologic response, imaging, immune reconstitution-like inflammation, graft or organ injury	(113–116, 120, 121)

**Table 6:** Representative clinical studies and registries informing checkpoint modulation in infectious diseases.

Disease setting	Representative study/registry	Checkpoint focus	Translational insight	Key caveat	Key references
HIV reservoir biology	NCT03239899; correlative studies	Pembrolizumab or PD-1 blockade	Reservoir and CNS/tissue signal assessment	Mechanistic endpoints; not cure efficacy	(9–12, 19, 20, 37, 55, 128, 129)
HIV with cancer or Kaposi sarcoma	NCT03767465; NCT05646082; cohorts	ICIs or dostarlimab with cART	Safety, feasibility, and immune monitoring in people living with HIV	Cancer context limits HIV-cure extrapolation	(53, 54, 122, 123)
AIDS-associated PML	NCT04091932	Pembrolizumab	Salvage PD-1 blockade in severe opportunistic viral disease	Highly selected setting	ClinicalTrials.gov identifier
Chronic HBV	NCT04465890	Subcutaneous anti-PD-L1 antibody ASC22	Infection-focused checkpoint modulation for HBV functional cure	Requires liver-safety and antigen-burden selection	(57, 58, 77, 131, 132, 134, 163, 164)
Chronic HBV combination therapy	NCT07573943	ASC22 plus peg-IFN-alpha and nucleos(t)ide analogs	Staged immune modulation on antiviral background therapy	Efficacy, sequencing, and hepatic safety unresolved	(57, 58, 131, 132, 134)
Severe sepsis or septic shock	NCT02576457; NCT02960854	BMS-936559 or nivolumab	Early safety, pharmacokinetic, and pharmacodynamic data	Not powered for mortality or enrichment strategy	(108, 109, 162, 167)
EBV-HLH or CAEBV	NCT04084626; NCT05039580	PD-1 antibody with or without lenalidomide	Checkpoint modulation in EBV hyperinflammation/lymphoproliferation	Interpret with HLH, HSCT, and GVHD risk	(73, 78, 86, 91, 161)
CAEBV and EBV-HLH biomarkers	NCT05841342	PD-1 response-predictor monitoring	Biomarker-oriented selection in EBV-driven disease	Observational; not efficacy testing	(73, 78, 91, 161)
Invasive mold infections	NCT06285188; case reports	Immunomonitoring or adjunctive checkpoint concepts	Immune phenotyping for future host-directed strategies	Registry is not ICI treatment; case reports hypothesis-generating	(120, 121, 125)

The available clinical evidence is best interpreted through its translational context. Ex vivo reversal of immune dysfunction and animal models establish biological plausibility and potential hazards, but they do not by themselves demonstrate clinical benefit. Oncology-adjacent cohorts provide useful feasibility and safety information, although they remain confounded by malignancy, prior therapies, corticosteroid exposure, tumor burden, and selection bias. Infection-directed early-phase trials are most informative when they include mechanistic endpoints, but they currently support primarily safety, pharmacodynamic activity, or preliminary efficacy rather than definitive infection-directed benefit.

For HIV, studies in people with HIV and cancer suggest that PD-1 and CTLA-4 blockade can be feasible in selected patients on ART. Cure-directed studies are now testing whether PD-1 blockade can affect immune nonresponse or reservoir biology (122, 123). For HBV, clinical development must balance antiviral immune restoration against liver injury. For EBV, checkpoint blockade is clinically relevant in EBV-associated malignancies, whereas non-malignant EBV disease remains investigational and potentially hazardous. For sepsis, early studies and preclinical data justify further development, but patient selection remains unresolved. For TB, reactivation during cancer ICI therapy makes broad therapeutic use unlikely without strong mechanistic justification.

Recent literature supports a precision-immunotherapy interpretation rather than a disease-agnostic model. Contemporary reviews argue that infectious-disease checkpoint blockade should be developed around pathogen-specific biology rather than borrowed wholesale from oncology (3, 124–126). In HIV, newer studies link checkpoint-associated cellular reservoirs, tolerogenic dendritic-cell interactions, and anti-PD-1^-^associated immune reprogramming to reservoir persistence or decay in selected patients (56, 76, 127–129). In HBV and HCV, practical management reviews increasingly emphasize screening, prophylaxis, and serial monitoring for viral reactivation or liver injury during ICI exposure (124, 125, 130–134). In EBV-associated disease, recent mechanistic and clinical reports clarify how latency programs, PD-L1 regulation, tumor biology, CAEBV, and EBV-HLH intersect with checkpoint responsiveness and inflammatory toxicity (72–81, 90, 135–137).

Across nonviral and acute infection settings, a similar pattern emerges: checkpoint biology is promising only when interpreted through pathogen, tissue, and host context. Sepsis reviews place checkpoint inhibition within a personalized immunotherapy landscape that includes immune profiling, enrichment strategies, cytokines, cellular therapies, and adaptive trial designs (75, 77–81, 138, 139). Mycobacterial literature highlights the tension between enhanced antimicrobial immunity and dysregulated inflammation during checkpoint blockade (93, 140). Malaria, fungal-infection, and broader T-cell exhaustion studies show that checkpoint and metabolic pathways can regulate pathogen-specific immunity but may also promote immunopathology or immune reconstitution-like inflammation (80, 115, 125, 130, 137, 141–145). Updated immune-related adverse event (irAE) and infection-management literature further stresses that suspected ICI toxicity must be distinguished from occult infection, reactivation, or treatment-related immunosuppression (146–148).

## 8 Safety: infection exacerbation, reactivation, and immune-related toxicity

Safety is integral to the therapeutic rationale in this field. Checkpoint blockade can shift the balance between pathogen control and inflammatory injury. Potential complications include latent infection reactivation, worsening active infection, immune reconstitution-like syndromes, cytokine-driven inflammation, and immune-related adverse events that mimic or obscure infection (149, 150).

Latent TB and HBV deserve particular attention. PD-1/PD-L1 blockade may destabilize immune containment of M. tuberculosis or increase hepatic immune activity in HBV-infected patients (77, 85, 98). Screening and prophylaxis should be adapted to local epidemiology and individual risk. In high TB-burden settings, baseline symptom assessment, prior TB history, chest imaging when indicated, and interferon-gamma release assay or tuberculin testing may be appropriate before cancer ICI therapy or infection-directed trials. For HBV, baseline HBsAg, anti-HBc, anti-HBs, HBV DNA, and liver function testing should be considered in at-risk patients, with antiviral prophylaxis or pre-emptive monitoring according to hepatology and oncology guidance. EBV requires a different framework because reactivation, lymphoproliferation, and inflammatory syndromes can overlap with malignancy, transplantation, and immune dysregulation.

Management of immune-related adverse events can paradoxically increase infection risk. Corticosteroids, TNF inhibitors, mycophenolate, and other immunosuppressive agents used to treat ICI toxicity may predispose patients to bacterial, viral, fungal, or opportunistic infections (151, 152). Thus, ICIs may enhance antimicrobial immunity in selected contexts while indirectly increasing infection risk through toxicity management. Infection surveillance should extend beyond the period of checkpoint blockade itself.

Immunocompromised and transplant populations require particular caution. Patients with hematologic malignancy, neutropenia, advanced HIV, recent chemotherapy, prolonged corticosteroid exposure, critical illness, solid-organ transplantation, or allo-HSCT may face overlapping risks of uncontrolled infection and excessive immune activation. In solid-organ transplant recipients, checkpoint blockade can precipitate graft rejection, whereas exposure before or after allo-HSCT may amplify alloreactive T-cell responses and increase GVHD risk (91, 150–152). These concerns are especially relevant for CAEBV, EBV-associated lymphoproliferation, refractory fungal infection, and post-transplant viral complications, where checkpoint modulation may be considered as salvage therapy but could destabilize graft or inflammatory control.

Diagnostic ambiguity is another practical challenge. Fever, pneumonitis, hepatitis, colitis, myocarditis, lymphadenopathy, and cytokine elevation may reflect infection, immune-related toxicity, pathogen reactivation, malignancy progression, or overlapping processes. Pneumonitis must be distinguished from bacterial pneumonia, TB, invasive fungal disease, or viral pneumonitis; hepatitis may represent immune-related hepatitis, HBV flare, drug injury, or infectious liver involvement; and colitis may overlap with cytomegalovirus or Clostridioides difficile infection. Multidisciplinary management involving infectious disease specialists, immunologists, oncologists, hepatologists, transplant physicians, and critical care clinicians is therefore essential.

In infection-directed ICI trials, safety monitoring should be built around anticipated failure modes. HIV studies require plasma HIV RNA monitoring, reservoir assays, inflammatory markers, and surveillance for immune activation. HBV trials require frequent liver enzymes, bilirubin, international normalized ratio, HBV DNA, and HBsAg kinetics. EBV studies require EBV DNA, infected-cell lineage when feasible, ferritin, soluble IL-2 receptor, cytopenias, liver function, and HLH assessment. Sepsis trials require real-time organ dysfunction scoring, secondary infection surveillance, cytokine monitoring, and clear stopping rules for inflammatory worsening. TB studies, if attempted, would require serial microbiology, imaging, symptom assessment, and careful distinction between paradoxical inflammatory reactions and uncontrolled infection.

## 9 Biomarkers and patient selection

The future of ICIs in infectious diseases will depend on patient selection. Single-marker approaches are unlikely to be sufficient (153, 154). PD-1 or PD-L1 expression alone cannot distinguish protective immune restraint from pathological exhaustion, nor can it identify reversible pathogen-specific T-cell dysfunction (155). PD-1 should therefore be interpreted together with TCF-1, TIM-3, antigen specificity, differentiation state, ligand distribution, and tissue context.

Useful biomarker frameworks should integrate four domains. The first is pathogen biology: pathogen burden, replication kinetics, reservoir location, antimicrobial susceptibility, and tissue tropism. The second is immune phenotype: lymphocyte count, monocyte HLA-DR, cytokine response capacity, checkpoint co-expression, T-cell receptor clonality, transcriptional exhaustion signatures, and the presence of progenitor-like exhausted T cells. The third is tissue vulnerability: liver reserve in HBV, lung damage in TB, organ dysfunction in sepsis, and CNS or hematologic involvement in EBV-related disease. The fourth is treatment context: concurrent antimicrobials, ART, antiviral therapy, chemotherapy, corticosteroids, transplant immunosuppression, and microbiome-disrupting antibiotics.

A practical biomarker strategy should distinguish routine clinical markers from assays that remain investigational. Clinically available markers include complete blood count with differential, absolute lymphocyte count, CD4^+^ T-cell count in HIV, plasma HIV RNA, HBV DNA, quantitative HBsAg where available, HCV RNA, EBV DNA, liver enzymes, bilirubin, albumin, international normalized ratio, renal function, C-reactive protein, procalcitonin, ferritin and triglycerides in HLH-like syndromes, imaging, microbiology, and standard serological screening. These tests are useful for baseline risk assessment and longitudinal safety monitoring, but they rarely establish checkpoint reversibility on their own.

Intermediate-feasibility assays include lymphocyte subset profiling, flow-cytometric checkpoint co-expression, monocyte HLA-DR, cytokine-release or ex vivo stimulation assays, and pathogen-specific T-cell assays. These approaches are realistic in tertiary centers and clinical trials, but they require standardization, fresh-sample logistics, and disease-specific interpretation. Research tools, including single-cell transcriptomics, spatial profiling, TCR sequencing, intact proviral DNA assays, intrahepatic immune profiling, and multiplex tissue imaging, can define mechanisms and response phenotypes but should not be presented as routine clinical requirements. A tiered model is most practical: routine markers support screening and safety monitoring, intermediate assays enrich trial populations, and exploratory tools serve as mechanistic substudies. Future trials should define a minimal deployable biomarker panel for enrollment and monitoring, while reserving high-dimensional assays for exploratory endpoints.

## 10 Therapeutic strategies beyond monotherapy

ICI monotherapy is unlikely to be the dominant strategy for infectious-disease applications. Combination and staged approaches are biologically more plausible.

In HIV, checkpoint blockade may be paired with ART, latency-reversing agents, therapeutic vaccines, broadly neutralizing antibodies, or adoptive cellular therapy. The goal would be to expose infected cells and provide immune effectors capable of eliminating them (156). In HBV, antigen-lowering strategies, nucleos(t)ide analogs, siRNA-based approaches, therapeutic vaccines, and checkpoint modulation may need to be sequenced to reduce hepatic risk (157). In EBV-associated malignancies, ICIs may be combined with chemotherapy, radiotherapy, adoptive EBV-specific T cells, or targeted agents (158–160). In chronic active EBV disease, cellular therapy or hematopoietic stem cell transplantation may remain central, while checkpoint blockade should be considered investigational (161).

In sepsis, ICIs may be combined with other immunostimulatory approaches such as IL-7, GM-CSF, interferon-gamma, or agents that restore antigen presentation. However, combination immune stimulation increases the need for careful phenotyping and stopping rules (162). In TB, any checkpoint-based host-directed therapy would likely require concurrent effective antimycobacterial treatment and close monitoring of inflammatory injury.

Risk-mitigation designs may reduce harm. These include short-course dosing, lower-dose regimens, reversible or locally delivered checkpoint modulators, bispecific approaches that target infected tissues, and therapies aimed at downstream immune restoration rather than broad checkpoint blockade. The guiding principle should be controlled immune recalibration rather than maximal immune activation.

## 11 Cost, access, and global applicability

Cost and implementation barriers will shape the realistic use of checkpoint modulation in infectious diseases. ICIs remain expensive, require specialized administration infrastructure, and depend on timely laboratory monitoring, imaging, toxicity recognition, and multidisciplinary care. These requirements may be difficult to sustain in low- and middle-income settings, where HIV, HBV, TB, sepsis, parasitic disease, and invasive fungal infection are often most prevalent and access to diagnostics, antimicrobials, antivirals, and critical-care resources may already be constrained.

For this reason, infection-directed ICI strategies should be developed only where they add value beyond optimized pathogen-directed therapy, vaccination, prophylaxis, source control, and supportive care. Global applicability would be improved by short-course or low-dose regimens, clear stopping rules, pragmatic biomarker panels based on clinically available tests, and trial designs that include cost, feasibility, and health-system readiness as implementation endpoints. In the near term, checkpoint modulation for infectious diseases is most likely to remain a tertiary-center or clinical-trial strategy; broader deployment will require durable benefit, manageable toxicity, and cost-effectiveness in the populations most affected by these infections.

## 12 Future perspectives

The next phase of this field should move beyond asking whether checkpoint blockade can enhance antimicrobial immunity. A more precise question is: in which infection, at which stage, in which tissue, and in which immune phenotype does checkpoint inhibition produce net clinical benefit?

Several priorities follow. First, infection-focused trials should define mechanistic endpoints alongside clinical outcomes. Viral load, bacterial burden, antigen levels, reservoir measures, immune-cell function, tissue injury, and quality of immune restoration should be assessed together. Second, trials should enrich for patients with plausible reversible immune dysfunction rather than enrolling broad diagnostic categories. Third, safety frameworks should account for infection reactivation, inflammatory injury, and immunosuppression used to manage ICI toxicity. Fourth, EBV-associated diseases should be separated into biologically distinct entities, including EBV-associated malignancy, chronic active EBV disease, post-transplant lymphoproliferative disease, and nonspecific EBV reactivation.

Finally, checkpoint biology should be integrated with broader host-directed therapy. In infectious diseases, success will rarely come from releasing a single inhibitory pathway. It will more likely come from combining pathogen-directed treatment with rational immune modulation, guided by biomarkers that identify when immunity needs support and when it needs restraint.

### 12.1 Trial design considerations

Future trials should avoid enrolling patients solely on the basis of pathogen diagnosis. Instead, eligibility should integrate pathogen control, immune phenotype, and tissue-risk assessment. For chronic viral infections, this may include suppressed or partially controlled viremia, reservoir or antigen measurements, virus-specific T-cell phenotyping, and exclusion of patients with high risk of inflammatory organ injury. For sepsis, inclusion criteria should prioritize persistent immunosuppression rather than early inflammatory shock alone. For TB and EBV-driven inflammatory disease, trials should be especially conservative because checkpoint pathways may maintain tissue-protective restraint.

Endpoints should also be disease-specific. HIV studies should measure inducible reservoir, intact proviral DNA, cell-associated RNA, viral rebound after analytical treatment interruption where ethically justified, and HIV-specific immune function. HBV trials should integrate HBsAg decline, HBV RNA, covalently closed circular DNA surrogates, ALT flares, liver synthetic function, and functional cure. EBV studies should separate EBV DNA kinetics, infected-cell lineage, inflammatory markers, lymphoma progression, and HLH-like events. Sepsis trials should combine immune restoration endpoints with organ dysfunction, secondary infection, ICU outcomes, and mortality.

The timing and duration of checkpoint blockade may be as important as the molecular target. Infection-directed use will likely require lower exposure, fewer doses, or reversible modulation compared with cancer therapy. Adaptive trial designs could permit early stopping for inflammatory toxicity, pathogen progression, or absence of immune response. Because ICIs can have delayed effects, follow-up should extend beyond immediate pathogen control and include late immune-related adverse events and opportunistic infections caused by immunosuppressive treatment of toxicity.

## 13 Conclusions

Immune checkpoint inhibitors offer a compelling but hazardous opportunity in infectious diseases. Their greatest promise lies in settings where persistent pathogens induce reversible immune dysfunction that cannot be corrected by antimicrobial therapy alone. HIV, HBV, EBV-associated diseases, and sepsis illustrate different versions of this possibility, whereas TB, malaria, viral hepatitis, and EBV-driven inflammatory syndromes illustrate the danger of disrupting protective immune restraint.

The field should resist direct transplantation of the oncology checkpoint-blockade paradigm into infectious diseases. In infection, inhibitory receptors are not simply obstacles to immunity; they are regulators of host survival. Progress will depend on precision targeting, rational combinations, careful timing, and rigorous safety monitoring. If developed thoughtfully, checkpoint modulation may become an important component of host-directed therapy for selected infectious diseases; if applied indiscriminately, it may amplify the immunopathology that checkpoints evolved to prevent.
